# Splitting smarter: Differential privacy for secure healthcare federated learning

**DOI:** 10.1038/s41598-025-27472-1

**Published:** 2025-12-11

**Authors:** Munirat Yetunde Onireti, Raj Mani Shukla, Tapadhir Das

**Affiliations:** 1 School of Computing and Information Science, Anglia Ruskin University, Cambridge, CB1 1PT United Kingdom; 2 Department of Computer Science, University of the Pacific, Stockton, CA 95211 USA

**Keywords:** Healthcare, Biomedical imaging, Federated learning, Differential privacy, Security, Computational biology and bioinformatics, Health care, Mathematics and computing

## Abstract

Split Federated Learning (SplitFed) has emerged as a decentralized method of training ML models that enables multiple healthcare parties to collaboratively share models without sharing their raw data. This method, however, is vulnerable to label inference attacks, which can compromise patient privacy. Previous research efforts have attempted to address the question. However, these works do not conduct a detailed vulnerability analysis of SplitFed against label inference attacks. Additionally, some of these efforts propose differential privacy (DP) as a solution; the works focus on distributed learning paradigms where labels used for training the model are available to the clients, which is not a practical assumption. To address this, in this paper, we investigate the vulnerability of SplitFed models to label inference attacks in biomedical imaging. We propose a solution that incorporates DP into SplitFed to protect against label inference attacks. Additionally, we also provide a detailed vulnerability analysis of SplitFed against label inference attacks specific to healthcare applications. Finally, we propose a DP-based method for mitigating label inference attacks against Split-Fed models. Results indicate the efficacy of the SplitFed model under multiple conditions and found that the label inference accuracy changes from $100\%$ (No-DP) to $0\%$ (with DP). This indicates that integration of DP offers a robust mechanism for protecting patient privacy. Additionally, the usage of Cauchy noise in DP provides the best protection out of all noise categories, with a label inference accuracy of 0% while Exponential noise was the worst, resulting in a label inference accuracy of 68%.

## Introduction

The application of Machine Learning (ML) has become a transformative force in the rapidly evolving field of healthcare technology, particularly in the analysis of medical imaging data. ML models can assist clinicians in diagnosing diseases by deciphering complex patterns within medical images. However, one of the challenges with conventional centralized ML training in healthcare is that sensitive user data must be gathered from multiple locations, leading to potential confidentiality risks. Additionally, local jurisdictional laws can also restrict this data collection^1^. To address this, Federated Learning (FL) emerged as a decentralized method of training ML models that enables multiple parties to collaboratively train a model without sharing their raw data. FL is a distributed privacy-preserving method of training an ML model that prevents unauthorized access to private data, and allows for model training across decentralized devices, preserving the privacy of individual health data^2,3^.

**Fig. 1:**
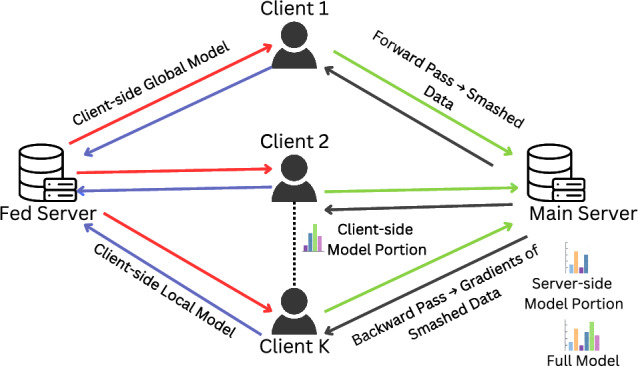
Architecture of a SplitFed framework.

The practice of FL faces several challenges, such as communication overhead and heterogeneity of participating devices/data^4^. To address these challenges, Split Federated Learning (SplitFed) was introduced. SplitFed offers an efficient solution by splitting the model between clients and the server. In SplitFed, the model is divided into multiple parts between the client and the server, and each part is trained on a different device. This approach enhances privacy and efficiency, as model weights are not shared between clients and the server. This also reduces the computational burden of the clients, as they only need to train a part of the model^2^. An illustration of SplitFed is provided in Fig. 1. In this figure, we note that there are *K* clients in the SplitFed setup. Each client contains the client-side portion of the model. The clients interact with the main server that contains the server-side portion of the model. The clients send over smashed data after doing a forward pass to the main server. The main server returns gradients of the smashed data to the clients after performing a backward pass. There is also the federated server that coordinates federated aggregation of the client-side models, by taking in the client-side local models as input and returning the client-side global models as output.

Despite the promises SplitFed shows in assisting with the communication overhead and heterogeneity of participating devices/data challenges of FL, it has been demonstrated to be compromised by cyber attacks that aim to compromise its functionality like^5–11^. Many times, these cyber attacks can occur from within the SplitFed setup and are carried out by malicious clients. This can put the architecture at risk, and can specifically jeopardize SplitFed models within the healthcare applications^12^. One specific attack that has been shown to severely compromise SplitFed models is the label inference attack^13,14^. In this attack, an adversary aims to infer the labels of the data used for training a model by interpreting subtle patterns within model updates. By doing so, they may be able to discover sensitive information^15^. This is especially problematic for SplitFed models within healthcare technology, as sensitive patient information can be leaked, resulting in privacy vulnerabilities. To date, there has been limited work to assess the robustness of SplitFed models against privacy vulnerabilities like label inference attacks.

To address this research gap, this paper analyses the robustness of SplitFed models against label inference attacks. Additionally, with a focus on label inference attacks, we aim to resolve the vulnerability of SplitFed models against label inference attacks using differential privacy (DP). DP is a technique that adds noise to the model parameters to prevent an attacker from inferring sensitive information about individual data points. The focus of this work is on healthcare applications, with a specific case study on brain tumor classification. Our major contributions are:

- Proposing a SplitFed-based technique for an FL-based Brain Tumour classification mechanism.
- Performing a detailed vulnerability analysis of SplitFed against label inference attacks to assess their security weaknesses.
- Developing a DP-based mechanism for mitigating label inference attacks in SplitFed models for FL-based Brain Tumor Classification.

The remainder of the paper is structured as follows. Section 2 introduces background work on SplitFed for healthcare. Section 3 talks about the methodology for our proposed work, while Sect. 4 presents the experimental findings. Finally, conclusions are drawn in Sect. 5.

## Related works

FL has emerged as a transformative approach at the pinnacle of ML and privacy preservation, specifically in the healthcare domain, where safeguarding patient data is imperative^16–18^. In^16^, the authors proposed a FL-enabled secure architecture for privacy-preserving in smart healthcare systems using blockchain as the privacy mechanism. Researchers in^17^ proposed a multi-tiered Federated Edge Aggregator to safeguard healthcare systems against privacy attacks. Authors in^18^ proposed an Edge-assisted FL framework to perform ML model retraining on healthcare data and protection against attacks. Applications of FL have also been seen in medical imaging^19–21^. Researchers in^19^ address the challenges of using deep learning in healthcare due to limited labeled data and the difficulty of sharing patient information across multiple centres. The work in^20^ presents the application of differentially private FL in the context of medical image analysis. Authors in^21^ introduce a variation-aware Fl framework to minimize image transformation variations into a common image space for medical images.

Despite the promises, some authors have focused on cyber attacks for FL in medical imaging applications^22–24^. Authors in^22^ discuss the importance of security and privacy in the context of FL in healthcare due to the sensitive nature of medical data. Researchers in^23^ provide a comprehensive survey that highlights several challenges related to privacy preservation in FL. The work in^24^ evaluated the impact of adversarial attacks on FL for medical image analysis. To protect against these, some researchers have investigated the usage of Split Learning (SL) and FL in medical imaging^25–27^. In^25^, the authors proposed SplitNN, which enabled the training of deep neural networks over multiple data sources in a distributed and secure manner while mitigating the need to share raw labeled data directly.^26^ discussed the concept of SL, a distributed deep learning method that enables health entities to collaboratively train deep learning models without sharing sensitive raw patient data. The work in^27^ introduces SplitFed as a novel approach that combines the strengths of FL and SL to address the limitations of both methods.

Despite the promises of SplitFed, researchers have found them vulnerable against label inference attacks^15,28^. Researchers in^28^ discuss the vulnerabilities of SpltFed to label inference attacks and evaluate the effectiveness of defence strategies against these attacks. The work in^15^ discusses UnSplit, a suite of attacks against SplitNN, a framework designed to address privacy concerns in distributed model training. To protect against this, the usage of DP has seen relevance in SplitFed models^27,29,30^. Discusses the use of DP as a protection method in the context of federated learning for healthcare applications.^30^ utilises DP to protect the privacy of client data in the context of FL.^27^ discusses the use of DP in the context of federated learning and splitfed learning. It describes how DP is applied to enforce strict privacy in the client-side model training algorithm, ensuring that individual examples do not have undue influence on the process of guaranteeing DP.

Despite the studied research efforts, there is a research gap in the literature. The literature has mainly focused on distributed learning paradigms where labels used for training the model are available to the clients. This is not a realistic scenario, as clients do not always necessarily contain the labels. Furthermore, there has been limited work on analysing the privacy vulnerabilities of SplitFed against label inference attacks and proposing measures to reduce their impact. A comprehensive analysis of the discussed existing research in this section, along with their comparison with the proposed research, is provided in the feature analysis table in Table 1.

**Table 1:** Feature Analysis Table for Related Work.

Work	FL	Healthcare application	Cybersecurity focus	SplitFed	Label inference attack	Differential privacy	Privacy analysis and countermeasure
^16^	$\checkmark$	$\checkmark$					
^17^	$\checkmark$	$\checkmark$					
^18^	$\checkmark$	$\checkmark$					
^19^	$\checkmark$	$\checkmark$					
^20^	$\checkmark$	$\checkmark$					
^21^	$\checkmark$	$\checkmark$					
^22^	$\checkmark$	$\checkmark$	$\checkmark$				
^23^	$\checkmark$	$\checkmark$	$\checkmark$				
^24^	$\checkmark$	$\checkmark$	$\checkmark$				
^25^	$\checkmark$	$\checkmark$	$\checkmark$	$\checkmark$			
^26^	$\checkmark$	$\checkmark$	$\checkmark$	$\checkmark$			
^27^	$\checkmark$	$\checkmark$	$\checkmark$	$\checkmark$			
^28^	$\checkmark$	$\checkmark$	$\checkmark$	$\checkmark$	$\checkmark$		
^15^	$\checkmark$	$\checkmark$	$\checkmark$	$\checkmark$	$\checkmark$		
^29^	$\checkmark$	$\checkmark$	$\checkmark$	$\checkmark$	$\checkmark$	$\checkmark$	
^30^	$\checkmark$	$\checkmark$	$\checkmark$	$\checkmark$	$\checkmark$	$\checkmark$	
**Our Work**	$\checkmark$	$\checkmark$	$\checkmark$	$\checkmark$	$\checkmark$	$\checkmark$	$\checkmark$

## Methods

**Fig. 2:**
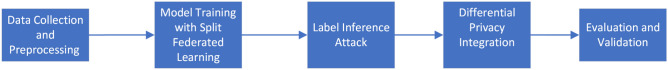
Methodology Flow Diagram.

This section introduces the proposed SplitFed technique for brain tumor classification, as illustrated in Fig. 2. In this framework, the first step is to conduct data collection and preprocessing. This can be performed using healthcare data samples and their corresponding labels. Next, we train the SplitFed model using the preprocessed data, which includes model initialization, data partitioning between clients and server, client-side training, server-side aggregation, global/client model updates, and model evaluations. After this, we conduct the label inference attack, which includes model training, gradient extraction, lone model initialization, prediction generation, label comparison, and iterative testing. Next, we incorporate DP as our defense mechanism, which includes noise addition to parameters and transmissions, noise generation, and parameter restoration. Finally, we evaluate the framework for efficacy at protecting against label inference attacks.

### Data collection and pre-processing

We start by loading our dataset, comprising MRI images of brain tumors and their corresponding labels. Before training, we pre-process the data by resizing the images to a uniform size, converting them to tensors, and normalizing their pixel values to a predefined range.

### Model training with split federated learning

**Fig. 3:**
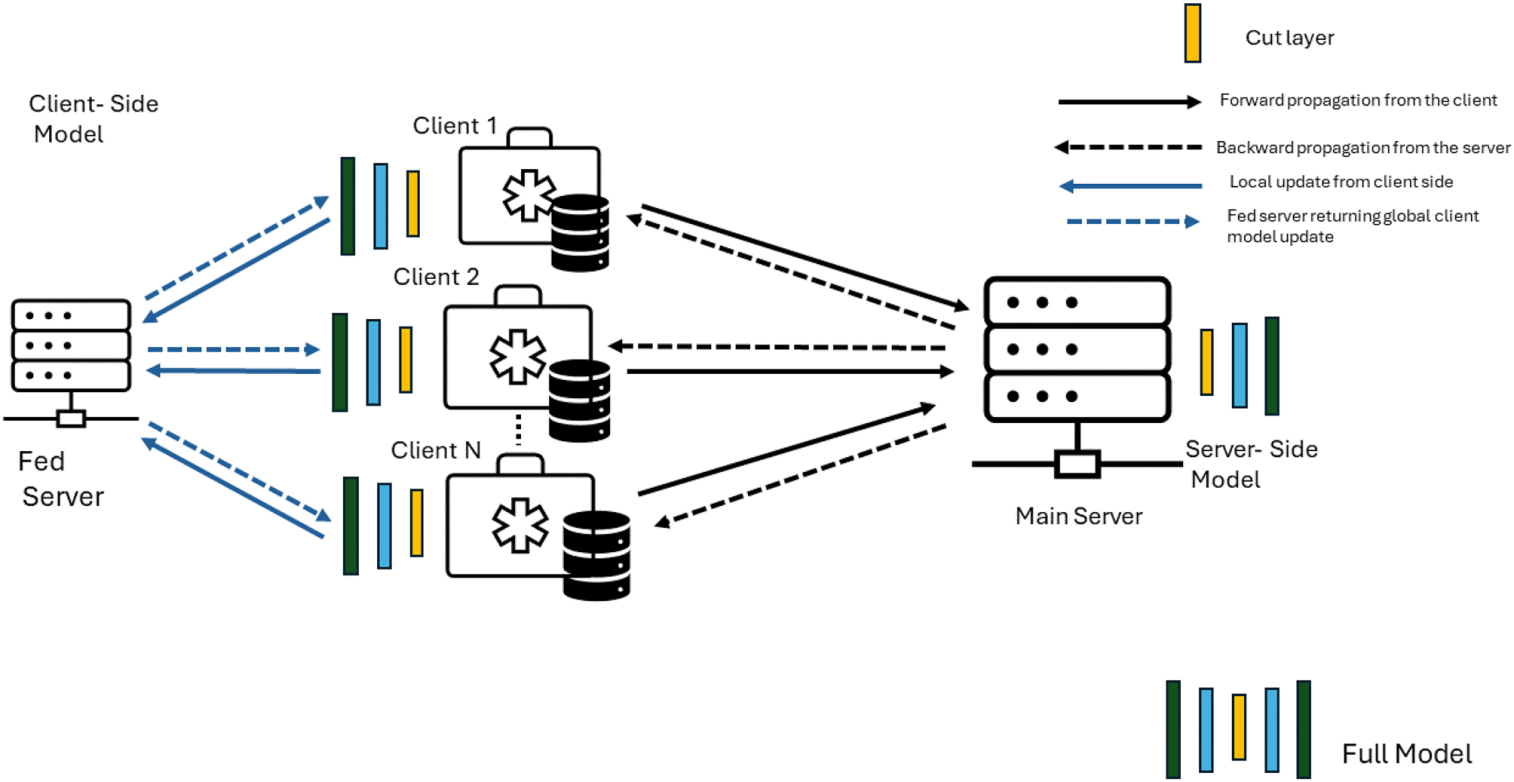
Proposed SplitFed Architecture.

Our proposed SplitFed model is illustrated in Fig. 3. Additionally, we also provide a tabular definition for the relevant variables in this research in Table 2.

**Table 2:** Methodology Variable Definitions.

Base Setup		Label Inference Attack		Differential Privacy Defense	
Variable	Definition	Variable	Definition	Variable	Definition
${\textbf{i}}$	Each client	$\mathbf {f_2}$	Client during Label Inference	$\varvec{param}$	Original model parameters
$\mathbf {x_i}$	Local dataset	$\mathbf {f_1}$	Server during Label Inference	$\varvec{param_{\text {noisy}}}$	Noisy model parameters
$\mathbf {Y_{c_i}}$	Client model	$\tilde{\mathbf {f_2}}$	Clone of $f_2$	$\varvec{noise}$	Sampled noise from specific distribution
$\mathbf {h_{Y_{c_i}}}$	Client model activations	${\tilde{z}}^*$	Inferred label	$\varvec{transmission}$	Original data transmission
${\textbf{k}}$	Total number of clients	$\varvec{\Phi _2}$	$f_2$ parameters	$\varvec{transmission_{\text {noisy}}}$	Noisy data transmission
$\mathbf {f_{Y_s}}$	Global model activation	$\tilde{\mathbf {f_2}}$	Parameters of $\tilde{f_2}$	$\varvec{\Theta ^t}$	Model parameters at time $t$
$\mathbf {Y_s}$	Global model input			$\varvec{\Theta ^{t+1}}$	Model parameters at time $t+1$
$\mathbf {L_i}$	Loss of $Y_{c_i}$			$\varvec{\eta }$	Learning rate
$\mathbf {y_i}$	Local labels			$\varvec{L_{\text {Local}}}$	Local loss function
				$\varvec{D_{\text {Local}}}$	Local dataset
				$\text {CauchyNoise}(\gamma , \sigma )$	Cauchy noise with scale $\sigma$ and location $\gamma$

***Model Initialization*** We initialize the client-side and server-side models. These models are architecturally identical and consist of convolutional layers followed by fully connected layers. The client and server models will participate in the collaborative training process.

***Data Partitioning*** The selected dataset is partitioned into multiple disjoint subsets, each corresponding to data held by different healthcare institutions or clients. Each client holds a local dataset comprising MRI images of brain tumors along with their corresponding labels.

***Client-Side Training*** Each client *i*, independently trains its local model using its local dataset $x_i$. Training is conducted using the dataloader object, which iterates through batches of data shuffled with each iteration. The client optimizer is used to update the parameters of the client model $Y_{c_i}$ based on the gradients computed during backpropagation. The computations of activations were conducted using:

1 $$\begin{aligned} h_{Y_{c_i}}(x_i), \text {for } i \in \{1, 2, \ldots , k\} \end{aligned}$$

After completing the forward pass through their local model, clients generate activations $h_{Y_{c_i}}(x_i)$ representing the output of the last layer. These activations are used as input to the server model for further processing.

***Server-Side Aggregation*** The server receives activations from all participating clients and aggregates them to reconstruct the global model’s activation output $(f_{Y_s}(h_{Y_{c_i}}(x_i)))$. The aggregated activations serve as input to the server-side model $Y_s$, allowing the server to perform computations on the combined information. With the aggregated activations, the server performs forward and backward propagation through its server-side model $Y_s$. This process computes gradients representing the direction and magnitude of parameter updates needed to improve the global model. The computed gradients are securely transmitted back to the respective clients for further processing.

### Server-side model updates

The Server-Side model updates can be denoted by:

2 $$\begin{aligned} Y_s \leftarrow Y_s - \eta \sum _{i=1}^{k} n_i \cdot \frac{\partial L_i}{\partial Y_{c_i}} \end{aligned}$$

***Client-Side Model Update*** Upon receiving the gradients from the server, each client updates its local model’s parameters based on the received gradients. The client optimizer is used to apply the gradients to the parameters of the client model $Y_{c_i}$, aligning it with the updates from the server. This iterative process of gradient descent ensures that all participating clients contribute to the collective improvement of the global model. This is represented by:

3 $$\begin{aligned} Y_{c_i} \leftarrow Y_{c_i} - \eta \sum _{i=1}^{k} n_i \cdot \frac{\partial {\mathcal {L}}_i}{\partial Y_{c_i}}, \quad \text {for } i \in \{1, 2, \ldots , k\} \end{aligned}$$

***Model Training and Loss Computation*** In each training iteration, we feed batches of images through the client model. The client model processes the images and passes the resulting activations to the server model. Using the activations received from the client model and the corresponding ground truth labels $y_i$, we compute the loss function $L_i$, which quantifies the disparity between the predicted and actual labels. The loss is computed using:

4 $$\begin{aligned} L_i(f_{Y_s}(h_{Y_{c_i}}(x_i)), y_i), \text {for } i \in \{1, 2, \ldots , k\} \end{aligned}$$

With the loss computed, we perform backpropagation to compute the gradients of the loss function $L_i$ for the model parameters. These gradients indicate the direction and magnitude of adjustments needed to minimize the loss.

***Final Model Update*** After obtaining gradients from the loss function, we update the parameters of the client $Y_{c_i}$ and server $Y_s$ models using an optimization algorithm. These updates aim to minimize the loss function $L_i$ and improve the models’ performance.

### Label infererence attack

Our proposed threat model is provided in 4a. Label inference attack is the type of attack where adversaries exploit model updates to infer sensitive information about individual data samples. The proposed threat model for SplitFed encompasses scenarios where malicious parties attempt to infer labels of data samples based on model updates exchanged during the training process. Adversaries may have access to gradient values, input data, and potentially even partial knowledge of the model architecture. The label inference attack aims to exploit model updates to infer sensitive information about individual data samples, particularly in the context of brain tumor classification using MRI images.

***Model Training*** The SplitFed model architecture comprises convolutional layers followed by max-pooling operations and fully connected layers for classification. Both the client $f_2$ and server $f_1$ models are trained using the Adam optimizer. The training data consists of brain MRI images pre-processed using standard transformations, including resizing and normalization.

***Gradient Extraction*** After training the SplitFed model, we extracted the gradients$f_2(f_1(x))$ of the model parameters associated with each client’s update. These gradients represent the sensitivity of the model’s parameters to changes in the input data.

***Lone Model Initialization*** A clone model ${\tilde{f}}_2$, identical to the original client model $f_2$, is initialised. This clone model serves as the adversary, attempting to infer the true labels of the input images based on the extracted gradients.

***Prediction Generation*** Using the gradients $f_2(f_1(x))$ obtained from the client model $f_2$ through backpropagation, the clone model generates predictions for each input image $x$. The predictions are based on the clone model’s weights and biases, which are adjusted iteratively during training to minimize the loss function $L$. The inferred labels are obtained using:

5 $$\begin{aligned} {\tilde{z}}^* = \arg \min _{{\tilde{z}} \in {\mathbb {Y}}} \text {MSE} \left( \frac{\partial L(f_2(f_1(x)), y) }{\partial \phi _2}, \frac{\partial L({\tilde{f}}_2(f_1(x)), {\tilde{z}})}{\partial {\tilde{\phi }}_2} \right) \end{aligned}$$

where:

- ${\tilde{z}}^*$represents the predicted label that minimizes the mean squared error (MSE).
- ${\mathbb {Y}}$ denotes the set of labels.
- $f_2(f_1(x))$ represents the output of the client model $f_2$ given the input $x$ processed by the first layer $f_1$.
- $y$ is the true label associated with the input $x$.
- $L$ denotes the loss function.
- $f_1$ is the server model
- $\Phi _2$ represents the parameters of the client model $f_2$.
- ${\tilde{f}}_2$ is the attacker’s clone model.
- ${\tilde{\Phi }}_2$ represents the parameters of the attacker’s clone model ${\tilde{f}}_2$.

***Label Comparison*** The predicted labels ${\tilde{z}}^*$ generated by the clone model are compared against the true labels $y$ of the input images $x$. This comparison allows us to evaluate the accuracy of the label inference process and assess the model’s vulnerability to inference attacks.

***Iterative Testing*** The label inference attack simulation is conducted iteratively on a subset of the test dataset. By testing the model on multiple samples, we obtain a more comprehensive understanding of its vulnerability to inference attacks across different scenarios and data distributions.

### Differential privacy

To protect against the label inference attacks, we propose the usage of differential privacy (DP) in our SplitFed training process. DP ensures that the output of the learning algorithm does not reveal information about any individual in the dataset. The integration of DP in SplitFed involves the addition of carefully calibrated noise to the model parameters during training. This noise obscures any patterns or information that could potentially lead to the identification of individual data points within the training dataset, thus safeguarding the privacy of each participant’s data. This noise is sampled from various distributions, including Gaussian, Laplace, Cauchy, and exponential, and its scale is adjusted to achieve the desired level of privacy while minimising the impact on model performance. Furthermore, we extend the DP protection to the data transmission process between clients and the server. By adding noise to the activations transmitted from clients to the server, we enhance the privacy of individual data points, mitigating the risk of information leakage during the training process.

***Noise Addition to Model Parameters*** During the training phase, noise is added to the model parameters to enhance privacy. This is achieved by introducing random noise sampled from a specified distribution and a scaling factor. The noise is added to each parameter of the model, thereby perturbing its values. By doing so, the sensitive information encoded in the model parameters is clouded, making it more challenging for adversaries to infer specific details about the training data or the model itself. This is denoted by:

6 $$\begin{aligned} param_{noisy}=param+noise \end{aligned}$$

where, $param_{noisy}$ represents the noisy model parameters, *param* represents the original model parameters, and *noise* represents the noise sampled from a specific distribution.

***Noise Addition to Data Transmissions*** Besides perturbing the model parameters, noise is introduced into the data transmissions between the client and the server during training. This involves augmenting the input data with random noise sampled from a predefined distribution and a scaling factor. The noisy input data undergoes forward propagation through the client model and backward propagation through the server model. Furthermore, noise is added to the gradients computed during backpropagation, ensuring that the information exchanged between the client and the server is protected against potential privacy breaches. This is represented as:

7 $$\begin{aligned} transmission_{noisy}=transmission+noise \end{aligned}$$

where $transmission_{noisy}$ represents the noisy data transmission, *transmission* represents the original data transmission (i.e. activations or gradients), and *noise* represents the noise added to the transmission.

***Noise Generation*** The equation  8 provides the mechanism for integrating DP into our proposed SplitFed architecture.

8 $$\begin{aligned} \theta ^{(t+1)} = \theta ^{(t)} - \eta \nabla L_{\text {local}}(\theta ^{(t)}, D_{\text {local}}) + \text {CauchyNoise}(\gamma , \sigma ) \end{aligned}$$

Where:

- $\theta ^{(t)}$ represents the model parameters at iteration $t$,
- $\theta ^{(t+1)}$ represents the updated model parameters at iteration $t+1$,
- $\eta$ is the learning rate,
- $L_{\text {local}}$ is the local loss function,
- $D_{\text {local}}$ is the local dataset,
- $\text {CauchyNoise}(\gamma , \sigma )$ represents Cauchy noise with scale parameter $\sigma$ and location parameter $\gamma$.

The Cauchy distribution has a location parameter $\gamma$ and a scale parameter $\sigma$. The probability density function (pdf) of the Cauchy distribution is given by:

9 $$\begin{aligned} f(x|\gamma ,\sigma ) = \frac{1}{\pi \sigma \left[ 1 + \left( \frac{x-\gamma }{\sigma }\right) ^2\right] } \end{aligned}$$

***Model Parameter Restoration*** To mitigate the potential accumulation of noise and ensure the stability of the model, a mechanism for restoring the initial parameters of the model was implemented. This mechanism, executed after each training iteration, helps maintain the integrity of the model parameters while still benefiting from the privacy-preserving effect of DP integration.

### Model evaluation

Periodic model evaluation is conducted on the test dataset to assess the performance of the trained model. Evaluation metrics, including the classification report, confusion matrix, and accuracy, are computed to gauge the effectiveness of the trained model in accurately classifying brain tumours.

## Experimental setup and results

**Table 3:** Overall Performance Metrics.

(a) Classification Report				
Class	Precision	Recall	F1-score	Support
0	0.93	0.90	0.92	300
1	0.94	0.88	0.91	306
2	0.99	1.00	0.99	405
3	0.91	0.99	0.95	300
**Accuracy**			**0.95**	**1311**
Macro Avg	**0.94**	**0.94**	**0.94**	**1311**
Weighted Avg	**0.95**	**0.95**	**0.95**	**1311**

(b) Attack Classification Report				
Class	Precision	Recall	F1-Score	Support
0	1.00	1.00	1.00	300
1	1.00	1.00	1.00	306
2	1.00	1.00	1.00	405
3	1.00	1.00	1.00	300
**Accuracy**			1.00	1311
Macro Avg	1.00	1.00	1.00	1311
Weighted Avg	1.00	1.00	1.00	1311

**Fig. 4:**
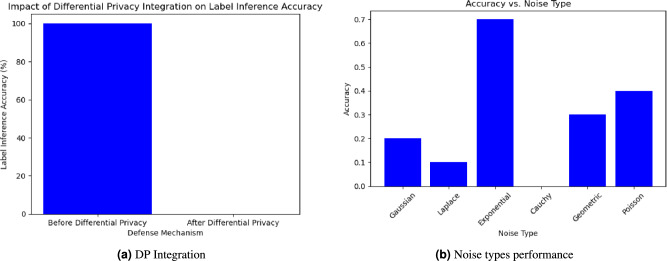
Experimental Results.

### Experimentation setup

***Setup*** Our proposed methodology is carried out using the Python Programming Language within the Jupyter Notebook environment. The dataset used was the Brain Tumour MRI Dataset^31^ comprises a collection of 7023 MRI images of various human brain tumours combined from three datasets: Figshare^32^, SARTAJ^33^ and Br35H^34^ dataset, which is classified into four classes: no-tumour (0), glioma (1), meningioma (2), and pituitary (3) tumour. For data pre-processing, we performed image resizing to fit the ML model, along with normalizing the data to a range of [-1, 1] using the min-max technique.

***Classification Model*** The BtmriNet architecture serves as the backbone of our SplitFed model for brain tumour classification. It is a convolutional neural network (CNN) designed to extract meaningful features from brain MRI images and classify them into relevant tumour categories. The architecture consists of multiple convolutional layers followed by max-pooling operations, a flattened layer, and fully connected layers for classification.

***Label Inference Attack Model*** The model to perform the label inference attack is the same model as the classification model. The only difference is that this model identifies the index of the candidate label with the lowest MSE loss. This index is then used to retrieve the corresponding label from the actual BtmriNet, which is returned as the inferred label.

***DP Integration*** In addition to the standard BtmriNet architecture, we introduce a variant called NoisyBtmriNet to enhance the robustness of the model against privacy attacks. NoisyBtmriNet incorporates DP mechanisms during both training and inference stages to mitigate privacy risks associated with SplitFed.

***Model Parameters*** Both BtmriNet and NoisyBtmriNet architectures are trained using the Adam optimizer with a learning rate of 0.001 and batch size of 64, and AMSGrad optimization. The optimizer and learning rate are based on empirical observations and experimentation to achieve optimal convergence and performance.

***Evaluation Metrics*** For performance analysis, we use standardized classification metrics: accuracy, precision, recall, f-measure, and label inference accuracy.

### Results

In this section, we present the results of our Splitfed model performance and the vulnerability assessment of the model, focusing on brain tumour classification using MRI images. We begin by assessing the model’s performance on the classification of MRI images, which are illustrated in Table 3a. We observe that the accuracy of the SplitFed model is 0.95. In addition, it achieved a macro-average precision score of 0.94, a macro-average recall score of 0.94, and a macro-average f-measure score of 0.94. Additionally, it achieved a weighted-average precision score of 0.95, a weighted-average recall score of 0.95, and a weighted-average f-measure score of 0.95. These high metrics indicate the model’s capability to accurately discern between different tumour classes, thereby aiding in precise diagnosis and treatment planning.

Next, we observe the vulnerability of the developed SplitFed model to label inference attacks. The primary metric used for assessment is label inference accuracy, which indicates the attacker’s ability to predict the correct labels of data samples based on model updates obtained during the SplitFed process. The results are provided in Table 3b. We observe that the label inference attack model achieved an accuracy of 1.0. In addition, it achieved a macro-average precision score of 1.0, a macro-average recall score of 1.0, and a macro-average f-measure score of 1.0. Additionally, it achieved a weighted-average precision score of 1.0, a weighted-average recall score of 1.0, and a weighted-average f-measure score of 1.0.This indicates that the attacker could accurately predict the labels of all evaluated data samples based on the information obtained from the SplitFed process. These results underscore the importance of implementing robust defence mechanisms to mitigate the risk of label inference attacks and enhance the security of SplitFed in healthcare applications.

Next, observe the performance of the SplitFed model against label inference attacks under DP. This is illustrated in Fig. 4a. We note that the integration of DP into SplitFed provided significant improvement in the defence against label inference attacks. Without DP, the label inference accuracy was observed to be $100\%$. After implementing DP, the label inference accuracy was $0\%$. The precision of 100% to 0% shows that the noise added through the DP mechanism effectively blocked sensitive information in the SplitFed process. This outcome demonstrates the efficacy of DP in providing strong privacy guarantees, as the attacker’s ability to infer labels from the model updates was effectively nullified.

Finally, we evaluate the achieved label inference accuracy under various noise types in DP, as shown in Fig. 4b. Among the implemented noise techniques, Cauchy noise resulted in a label inference accuracy of $0\%$, indicating that the attacker was unable to predict any labels correctly. In contrast, the least effective noise was Exponential noise, which resulted in a label inference accuracy of $68\%$. Cauchy noise proved most effective at reducing label inference accuracy due to its heavy-tailed nature^35^, which can produce large, unpredictable changes in model outputs. These strong perturbations disrupt the relationship between outputs and true labels, preventing attackers from reliably inferring them. Consequently, label inference becomes extremely difficult. However, it should also be noted that the large perturbation of the Cauchy nose can also affect model performance for datasets requiring precise output. On the other hand, noise types like Gaussian have a bell-shaped curve and produce small, predictable perturbations^36^. The exponential noise performs worst because the limited magnitude of the noise and one-sided nature mean that the attacker can often adjust for the bias or detect the trend in outputs, so label inference remains feasible as compared to other noise types^37^.

### Conclusion and future work

In this paper, we have addressed a gap in privacy-preserving ML for healthcare applications by investigating the effectiveness of SplitFed against label inference attacks. The methodology investigates the usage of DP as a way to protect against label inference attacks. We evaluate the efficacy of the SplitFed model under multiple conditions and found that the label inference accuracy changes from $100\%$ (No-DP) to $0\%$ (with DP). This indicates that integration of DP offers a robust mechanism for protecting patient privacy. Additionally, the usage of Cauchy noise in DP provides the best protection out of all noise categories. For future work, we will explore more advanced privacy mechanisms to enhance the privacy guarantees of SplitFed systems. We will also perform longitudinal data analysis and evaluation of the long-term impact of SplitFed on patient outcomes and healthcare delivery. In addition, we will also investigate the time and space complexity of the proposed framework in practical deployment environments. Finally, we will also perform a comprehensive benchmark of the benchmark downstream performance metrics for the proposed framework, targeting multiple healthcare datasets in various applications like cancer diagnosis and neurological disorder identification.

## Data Availability

The brain tumor MRI dataset used in this study is publicly available on Kaggle at https://www.kaggle.com/datasets/masoudnickparvar/brain-tumor-mri-dataset.
